# Efficacy of argon–helium cryoablation combined with PD-1 inhibitors in non-small cell lung cancer

**DOI:** 10.2340/1651-226X.2025.44215

**Published:** 2025-11-12

**Authors:** Zheng Zheng, Bo Tian, Yonghui An, Wei Wang, Miaomiao Zhang, Wenhua Ma, Ying Guo, Yao Fan, Na Li

**Affiliations:** Department of Oncology, the First Hospital of Hebei Medical University, Shijiazhuang, Hebei, China

**Keywords:** Argon–helium cryoablation, PD-1 inhibitor, non-small cell lung cancer, randomized controlled trial, efficacy, immune function

## Abstract

**Background and purpose:**

This study aimed to evaluate the efficacy and safety of argon–helium cryoablation combined with Programmed Death-1 (PD-1) inhibitors versus PD-1 inhibitors plus chemotherapy in treating non-small cell lung cancer (NSCLC).

**Patient/material and methods:**

In this single-center, open-label, randomized controlled trial, 60 NSCLC patients treated between December 2020 and December 2023 were enrolled. Patients were randomly assigned (1:1) to either a study group (argon–helium cryoablation + PD-1 inhibitor, *n* = 30) or a control group (PD-1 inhibitor + chemotherapy, *n* = 30). Allocation was concealed using sequentially numbered, opaque, sealed envelopes (SNOSE). Primary endpoints were overall survival (OS) and progression-free survival (PFS). Secondary endpoints included short-term efficacy – objective response rate (ORR), disease control rate (DCR) – immune function changes (CD4+, CD8+, CD4+/CD8+), and adverse reactions, assessed after four cycles and during a 1-year follow-up.

**Results:**

ORR and DCR were higher in the study group (ORR: 73.33% vs. 53.33%; DCR: 90.00% vs. 83.33%), though not statistically significant (*P* > 0.05). Baseline immune parameters were similar. After four cycles, the study group showed statistically significantly higher CD4+ and CD4+/CD8+ ratios, and lower CD8+ levels (all *P* < 0.001). Adverse reactions were comparable between groups (*P* > 0.05). At 1-year follow-up, the PFS rate was 63.3% vs. 43.3%. The study group had a statistically significantly better OS (median not reached vs. 10.3 months, *P* = 0.003) and longer median PFS (9.6 vs. 8.3 months, *P* = 0.005).

**Interpretation:**

Argon–helium cryoablation combined with PD-1 inhibitors statistically significantly improved OS, PFS and immune function in NSCLC patients, offering a promising alternative to standard therapy.

## Graphical abstract

**Figure:**
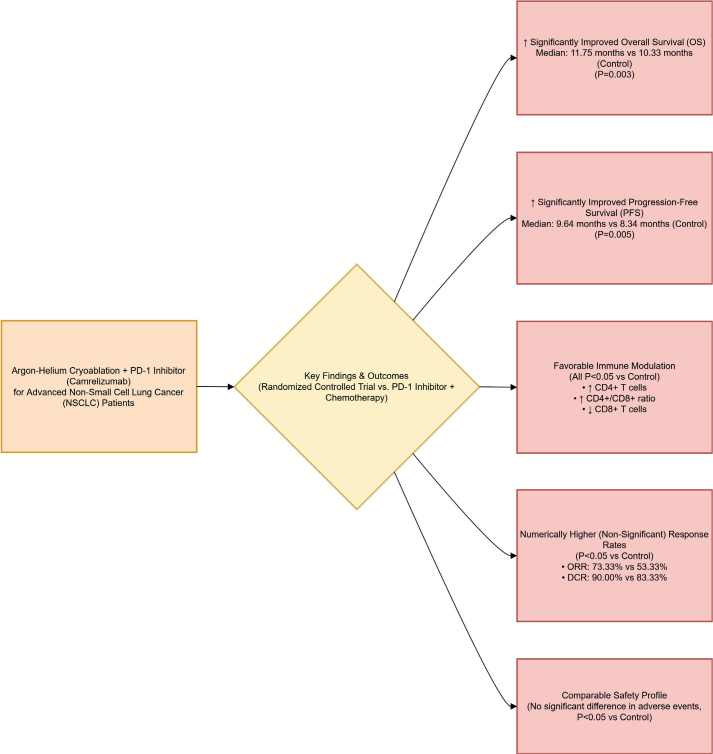

## Introduction

Lung cancer remains a global health challenge, ranking as the second most commonly diagnosed cancer and the leading cause of cancer-related mortality worldwide [1]. In China, lung cancer exhibits the highest incidence and mortality rates among all malignancies. According to recent statistics from the National Cancer Center, approximately 828,000 new cases are diagnosed annually in China, with around 657,000 deaths reported each year [2, 3]. Histologically, non-small cell lung cancer (NSCLC) comprises approximately 85% of primary lung cancers [4]. A significant proportion of NSCLC patients, estimated at around 50%, present with advanced-stage disease at initial diagnosis, precluding them from curative surgical resection [5]. Furthermore, while traditional thoracotomy can effectively remove tumor lesions, it is associated with substantial surgical trauma and an increased risk of postoperative complications.

In recent years, minimally invasive local ablative techniques have gained prominence. Argon–helium cryoablation, a thermal ablation modality, has been widely applied in the treatment of various malignancies, including NSCLC, liver cancer and pancreatic cancer [6]. This technique can achieve radical ablation in early-stage lung cancer patients ineligible for surgery and can cytoreduce primary or metastatic tumors in advanced-stage disease, thereby alleviating local symptoms. Its utility is recognized in guidelines such as those from the National Comprehensive Cancer Network (NCCN), particularly for the local management of early-stage NSCLC in patients who are not candidates for surgery, or for controlling oligometastatic or oligoprogressive disease in advanced stages [7]. Cryoablation induces tumor cell death through rapid freezing and thawing cycles, leading to ice crystal formation, osmotic shifts and vascular damage within the tumor [8]. However, its application can be limited by tumor location, particularly for lesions adjacent to major blood vessels, which can create a ‘heat sink’ effect, and it carries risks such as pneumothorax and hemorrhage [9].

Concurrently, immunotherapy, particularly the use of immune checkpoint inhibitors (ICIs) targeting the Programmed Death-1 (PD-1)/Programmed Death Ligand-1 (PD-L1) pathway, has revolutionized the treatment landscape for advanced cancers [10]. Camrelizumab, a humanized anti-PD-1 IgG4 monoclonal antibody, has been approved as a standard treatment for advanced NSCLC and works by blocking the PD-1/PD-L1 interaction, thereby reinvigorating the host’s T-cell-mediated anti-tumor immune response [11]. This blockade is intended to enhance the anti-tumor activity of cytotoxic T lymphocytes (CTLs) (CD8+) and modulate the function of helper T cells (CD4+), which are critical components of cellular immunity. The relative balance of these T cell subsets, often represented by the CD4+/CD8+ ratio, is a key indicator of immune status and can be influenced by effective cancer therapies. However, the efficacy of PD-1 inhibitor monotherapy or its combination with chemotherapy can be limited, with relatively low objective response rates (ORRs) in unselected populations, necessitating exploration of combination strategies to enhance therapeutic outcomes [12, 13].

The combination of cryoablation with PD-1 inhibitors presents a theoretically synergistic approach. Cryoablation can lead to the release of tumor-specific antigens from ablated cancer cells, potentially eliciting a systemic anti-tumor immune response (an ‘abscopal effect’) when combined with immunotherapy [14, 15]. This local tumor destruction and subsequent antigen release might prime the immune system, making tumors more susceptible to PD-1 blockade by enhancing T-lymphocyte-mediated killing of malignant cells. Such a combination could offer a more potent therapeutic strategy for advanced NSCLC. Early-phase and retrospective studies have suggested that combining cryoablation with ICIs can yield synergistic effects and is well-tolerated in patients with advanced NSCLC [16]. Despite this promising rationale, there is limited robust clinical evidence from randomized controlled trials directly comparing argon–helium cryoablation plus PD-1 inhibitors against standard chemo-immunotherapy regimens in NSCLC. Therefore, the present study was conducted to rigorously evaluate the efficacy and safety of argon–helium cryoablation combined with PD-1 inhibitors versus a standard chemo-immunotherapy regimen in patients with NSCLC.

## Patients/material and methods

### Study design and ethical considerations

This was a single-center, open-label, parallel-group, randomized controlled study conducted at a tertiary academic medical center in China. The study protocol was designed in accordance with the principles of the Declaration of Helsinki and the CONSORT 2010 guidelines [17]. Approval was obtained from the Ethics Committee of our hospital, and all participating patients provided written informed consent prior to any study-related procedures.

### Patient population and eligibility criteria

Patients diagnosed with NSCLC and treated between December 2020 and December 2023 were prospectively screened. Inclusion criteria were: (1) Pathologically confirmed NSCLC, stage IIIB, IIIC, or IV (AJCC 8th Edition); (2) At least one measurable tumor lesion according to Response Evaluation Criteria in Solid Tumors version 1.1 (RECIST 1.1) (18); (3) Estimated survival time ≥ 6 months; (4) Receiving the assigned study regimen for the first time; (5) Unable or unwilling to undergo surgical resection; (6) Karnofsky Performance Status (KPS) score ≥60; (7) Conscious and capable of effective communication; (8) Eastern Cooperative Oncology Group (ECOG) performance status score of 0–2. Exclusion criteria were: (1) Presence of other active malignant tumors; (2) Significant uncontrolled hepatic (total bilirubin > 1.5 × ULN, AST/ALT > 2.5 × ULN or > 5 × ULN if liver metastases present) or renal dysfunction (creatinine clearance < 50 mL/min); (3) Non-primary NSCLC; (4) Known allergy or contraindication to study drugs; (5) Coexisting active tuberculosis, uncontrolled systemic infections or other significant pulmonary diseases that would interfere with treatment or outcome assessment; (6) Pregnancy or lactation; (7) Severe psychiatric disorders; (8) Uncorrected coagulation disorders (e.g. INR > 1.5 or platelet count < 75 × 10^9^/L).

### Randomization and blinding

Eligible patients who provided informed consent were randomly assigned in a 1:1 ratio to either the study group or the control group. A computer-generated random number table was used for simple randomization. The allocation sequence was concealed using sequentially numbered, opaque, sealed envelopes (SNOSE), which were prepared by an independent statistician not involved in patient recruitment or treatment. The treating physician opened the next available envelope only after a patient was confirmed eligible and had provided informed consent. This was an open-label study; due to the distinct nature of the interventions, blinding of patients and care providers was not feasible. However, outcome assessors for radiological response (ORR, DCR, PFS) were blinded to the treatment allocation during centralized image review. Laboratory personnel assessing immune function markers were also blinded to group assignment during sample analysis.

### Treatment protocols

#### Control group

Patients received PD-1 inhibitor (Camrelizumab) at a dose of 200 mg intravenously every 3 weeks (q3w), which is the standard approved dosage [19]. This was combined with standard platinum-based doublet chemotherapy consisting of either: (1) For non-squamous NSCLC: Pemetrexed 500 mg/m² intravenously on day 1 of each 3-week cycle, plus Carboplatin intravenously on day 1, dosed to achieve an Area Under the Curve (AUC) of 5 mg/mL·min; or (2) For squamous NSCLC: Gemcitabine 1250 mg/m² on days 1 and 8, plus Carboplatin AUC 5 on day 1. Treatment was administered for a total of four cycles.

#### Study group

Patients received argon–helium cryoablation followed by PD-1 inhibitor therapy. The PD-1 inhibitor regimen (Camrelizumab 200 mg IV q3w for four cycles) was identical to the control group.

#### Argon–helium cryoablation procedure

**Figure 1:**
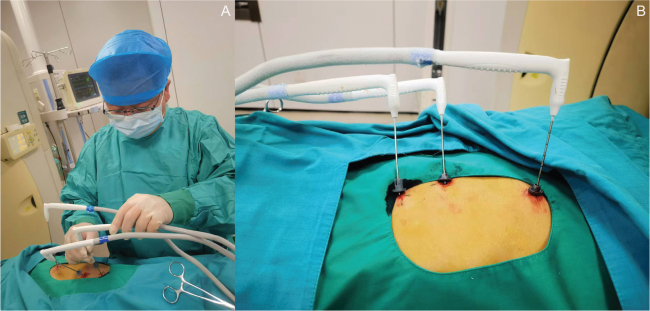
Representative Images of the Argon–Helium Cryoablation Procedure. (A) The physician performing CT-guided percutaneous puncture and insertion of cryoprobes into the tumor. (B) Multiple cryoprobes inserted into the target lesion during the freezing phase of cryoablation, aiming to create an ice ball that encompasses the tumor and a margin.

Performed within 7 days before the first PD-1 inhibitor dose. Patients were placed in a position depending on tumor location. After local infiltration anesthesia, a 0.5 cm skin incision was made. Under CT guidance, the cryoprobe(s) (1.7 mm or 2.4 mm diameter) were rapidly inserted into the target tumor, based on pre-procedural imaging for direction, angle and depth (Figure 1A). The goal was to encompass the tumor and a 5–10 mm margin with the ice ball formed during freezing (Figure 1B). The cryosurgical system was activated for two freeze–thaw cycles. Each rapid freeze cycle aimed to achieve a target temperature between –135°C and –145°C for 15–20 min, followed by passive or active thawing until the temperature rose to 0°C. After the second thaw, when the cryoprobe(s) could be easily detached, they were withdrawn. Hemostatic absorbable gelatin sponge was packed into the probe track. Post-procedural care included continuous monitoring for at least 6 h, prophylactic hemostatic agents for 3 days and a chest X-ray 24 h post-procedure.

### Outcome measures and assessments

The primary endpoints were Overall Survival (OS), defined as the time from randomization to death from any cause, and Progression-Free Survival (PFS), defined as the time from randomization to disease progression (RECIST 1.1) or death from any cause. Secondary endpoints included the following facts discussed further in the text.

#### Short-term efficacy

Tumor response was evaluated by blinded independent central review after four cycles of treatment (approximately 12 weeks from randomization) and then every 6–8 weeks, according to the modified Response Evaluation Criteria in Solid Tumors (mRECIST) [20]. Responses were categorized as Complete Response (CR), Partial Response (PR), Stable Disease (SD) or Progressive Disease (PD). The ORR was defined as (CR + PR), and the Disease Control Rate (DCR) was (CR + PR + SD).

#### Immune function markers

Peripheral venous blood was collected at baseline and after four cycles of therapy. Cluster of Differentiation 4+ (CD4+) and Cluster of Differentiation 8+ (CD8+) T lymphocyte subsets were quantified using a CytoFLEX flow cytometer. The CD4+/CD8+ ratio was calculated.

#### Adverse events

Adverse events (AEs) were recorded and graded according to the National Cancer Institute Common Terminology Criteria for AEs version 5.0 (NCI-CTCAE v5.0) [21]. Monitoring included physical examinations, vital signs and laboratory tests at baseline and before each treatment cycle.

#### Long-term efficacy

Patients were followed up every 3 months after completion of the four treatment cycles for survival and disease status for up to 1 year from randomization. The 1-year PFS rate was also calculated.

### Sample size calculation

The sample size was calculated based on the primary endpoint of median PFS. Based on previous studies and institutional data, the median PFS for patients receiving PD-1 inhibitors plus chemotherapy was estimated to be approximately 8 months [19, 22]. We hypothesized that argon–helium cryoablation combined with PD-1 inhibitors could improve median PFS to 11 months. Using a log-rank test with a two-sided significance level (alpha) of 0.05 and 80% power, a sample size of approximately 28 patients per group would be required. To account for potential dropouts or data incompleteness (< 10%), the target enrollment was set to 30 patients per group, yielding a total sample size of 60 patients.

### Statistical analysis

All efficacy analyses were performed on the intention-to-treat (ITT) population, defined as all randomized patients. Safety analyses included all patients who received at least one dose of study treatment. Data were analyzed using SPSS version 27.0. Continuous data were tested for normality using the Shapiro–Wilk test. Normally distributed data were expressed as mean ± standard deviation (SD) and compared using independent samples *t*-tests. Non-normally distributed data were presented as median (interquartile range [IQR]) and compared using the Mann–Whitney U test. Categorical data were expressed as counts (percentages) and compared using the Chi-square test or Fisher’s exact test, as appropriate. OS and PFS were estimated using the Kaplan–Meier method, and survival curves were compared using the log-rank test. Hazard ratios (HRs) and their 95% confidence intervals (CIs) were calculated using Cox proportional hazards models. A two-sided *P* < 0.05 was considered statistically significant.

## Results

### Patient enrollment flow and baseline characteristics

Between December 2020 and December 2023, a total of 73 patients with NSCLC were assessed for eligibility. Of these, 13 patients were excluded (two due to sepsis, two with COPD, one with systemic sclerosis, two with Sjogren’s syndrome, one with rheumatoid arthritis, one with concomitant gastric cancer, one with liver cancer, two with viral hepatitis and one with drug-induced liver injury). The remaining 60 eligible patients were randomized into two groups (*n* = 30 each): the study group and the control group. All 60 randomized patients received their allocated interventions, completed the planned four cycles of systemic therapy, and were followed for at least 1 year or until death/progression. No patients were lost to follow-up or discontinued the intervention prematurely. All 60 patients were included in the ITT analysis. The trial was concluded as planned. The enrollment process is summarized in the CONSORT flow diagram (Supplementary Figure 1). The baseline demographic and clinical characteristics were well-balanced between the two groups, with no clinically meaningful differences observed (Table 1).

**Table 1:** Comparison of baseline characteristics between the two groups.

Characteristic	Study group (*n* = 30)	Control group (*n* = 30)
**Gender (male/female)**, *n* (%)	17 (56.7) / 13 (43.3)	18 (60.0) / 12 (40.0)
**Age (years)**, mean ± SD	53.3 ± 12.0	53.0 ± 12.7
**BMI (kg/m²)**, mean ± SD	22.7 ± 3.8	22.6 ± 3.5
**Disease duration (months)**, mean ± SD	3.3 ± 1.1	3.1 ± 1.0
**Number of tumors**, *n* (%)		
1	5 (16.7)	6 (20.0)
≥2	25 (83.3)	24 (80.0)
**Smoking history (yes)**, *n* (%)	22 (73.3)	20 (66.7)
**Alcohol consumption (yes)**, *n* (%)	7 (23.3)	5 (16.7)
**Tumor location**, *n* (%)		
Central	19 (63.3)	18 (60.0)
Peripheral	11 (36.7)	12 (40.0)
**Pathological type**, *n* (%)		
Adenocarcinoma	16 (53.3)	17 (56.7)
Squamous cell carcinoma	14 (46.7)	13 (43.3)
**Clinical stage**, *n* (%)		
IIIB	15 (50.0)	16 (53.3)
** **IIIC	9 (30.0)	11 (36.7)
IV	6 (20.0)	3 (10.0)

### Comparison of short-term efficacy

After four cycles of treatment, the ORR in the study group was 73.33% (22/30; 95% CI: 54.1–87.7%; CR: 6 [20.00%], PR: 16 [53.33%]) and in the control group was 53.33% (16/30; 95% CI: 34.3–71.7%; CR: 3 [10.00%], PR: 13 [43.33%]). The DCR was 90.00% (27/30; 95% CI: 73.5–97.9%; SD: 5 [16.67%]) in the study group and 83.33% (25/30; 95% CI: 65.3–94.4%; SD: 9 [30.00%]) in the control group. While both ORR and DCR were numerically higher in the study group, these differences did not reach statistical significance (ORR difference: 20.00%, 95% CI: –4.09% to 44.09%, *P* = 0.108; DCR difference: 6.67%, 95% CI: –10.05% to 23.38%, *P* = 0.447) (Table 2).

**Table 2:** Comparison of short-term efficacy between the two groups n (%).

Response	Study group (*n* = 30)	Control group (*n* = 30)	*P*
Complete Response (CR)	6 (20.00)	3 (10.00)	
Partial Response (PR)	16 (53.33)	13 (43.33)	
Stable Disease (SD)	5 (16.67)	9 (30.00)	
Progressive Disease (PD)	3 (10.00)	5 (16.67)	
**Objective Response Rate (ORR)(CR+PR)**	**22 (73.33)**	**16 (53.33)**	0.108
**Disease Control Rate (DCR)(CR+PR+SD)**	**27 (90.00)**	**25 (83.33)**	0.447

### Comparison of immune function

At baseline, there were no statistically significant differences in peripheral blood CD4+ T cell counts, CD8+ T cell counts, or the CD4+/CD8+ ratio between the study and control groups (all *P* > 0.05). After four cycles of treatment, the study group showed statistically significantly higher mean CD4+ T cell counts (40.9 ± 2.7 vs. 34.3 ± 2.5 cells/μL; mean difference 6.6 cells/μL, 95% CI: 5.0–8.1) and a statistically significantly higher CD4+/CD8+ ratio (2.9 ± 1.0 vs. 1.5 ± 0.9; mean difference 1.3, 95% CI: 0.9–1.8) compared to the control group. Conversely, the mean CD8+ T cell count was statistically significantly lower in the study group (14.2 ± 2.7 vs. 22.3 ± 2.8 cells/μL; mean difference –8.1 cells/μL, 95% CI: –9.6 to –6.5) compared to the control group (Table 3).

**Table 3:** Comparison of immune function parameters between the two groups (mean ± SD).

Parameter	Timepoint	Study group (*n* = 30)	Control group (*n* = 30)	Mean difference (95% CI)	*P*
**CD4+ (cells/μL)**	Baseline	32.2 ± 2.5	32.3 ± 2.5	N/A	0.928
	After 4 cycles	40.9 ± 2.7	34.3 ± 2.5	6.6 (5.0 to 8.1)	< 0.001
**CD8+ (cells/μL)**	Baseline	25.7 ± 2.7	25.8 ± 2.7	N/A	0.884
	After 4 cycles	14.2 ± 2.7	22.3 ± 2.8	-8.1 (-9.6 to -6.5)	< 0.001
**CD4+/CD8+ ratio**	Baseline	1.3 ± 1.0	1.3 ± 0.9	N/A	1.000
	After 4 cycles	2.9 ± 1.0	1.5 ± 0.9	1.3 (0.9 to 1.8)	< 0.001

### Comparison of adverse reactions

All 60 patients were included in the safety analysis. The incidence and severity of treatment-related adverse reactions were generally comparable between the two groups (Table 4). The most common AEs included abdominal pain/diarrhea, decreased appetite, dyspnea, fatigue/dizziness, nausea/vomiting, rash, thrombocytopenia and leukopenia. The overall incidence of Grade 1 AEs was 53.33% (16/30) in the study group and 60.00% (18/30) in the control group (*P* = 0.603). The overall incidence of Grade 2 AEs was 36.67% (11/30) in the study group and 50.00% (15/30) in the control group (*P* = 0.297). No Grade 4 or 5 AEs were reported. Grade 3 AEs were infrequent: in the study group, one patient experienced Grade 3 leukopenia (3.3%); in the control group, one patient experienced Grade 3 leukopenia (3.3%) and one experienced Grade 3 thrombocytopenia (3.3%). No Grade 3 or higher AEs were directly attributable to the cryoablation procedure. Immune-related adverse events (irAEs) were noted, with one patient in the control group experiencing Grade 3 pneumonitis that required treatment discontinuation and high-dose corticosteroids. Most other irAEs were Grade 1–2 and managed with supportive care or corticosteroids as per guidelines.

**Table 4:** Comparison of adverse reactions between the two groups n (%).

Adverse reaction	Grade	Study group (*n* = 30)	Control group (*n* = 30)	*P* *
Abdominal pain/diarrhea	I/II	3 (10.0)	6 (20.0)	0.291
Decreased appetite	I/II	3 (10.0)	4 (13.3)	1.000
Dyspnea	I/II	2 (6.7)	4 (13.3)	0.672
Fatigue/dizziness	I/II	1 (3.3)	3 (10.0)	0.612
Nausea/vomiting	I/II	3 (10.0)	4 (13.3)	1.000
Thrombocytopenia	I/II	4 (13.3)	6 (20.0)	0.732
	III	0 (0.0)	1 (3.3)	
Leukopenia	I/II	5 (16.7)	3 (10.0)	0.702
	III	1 (3.3)	1 (3.3)	
*Immune-Related Adverse Events (irAEs)*				
Rash/dermatitis	I/II	3 (10.0)	3 (10.0)	1.000
Pneumonitis	I/II	1 (3.3)	2 (6.7)	1.000
	III	0 (0.0)	1 (3.3)	
Hepatitis (ALT/AST elevation)	I/II	2 (6.7)	2 (6.7)	1.000
**Total Grade I AE Incidence**		**16 (53.33)**	**18 (60.00)**	**0.603**
**Total Grade II AE Incidence**		**11 (36.67)**	**15 (50.00)**	**0.297**

Note:

* *P*-values from Fisher’s exact test for any grade comparison unless specified.

### Comparison of long-term survival

**Figure 2:**
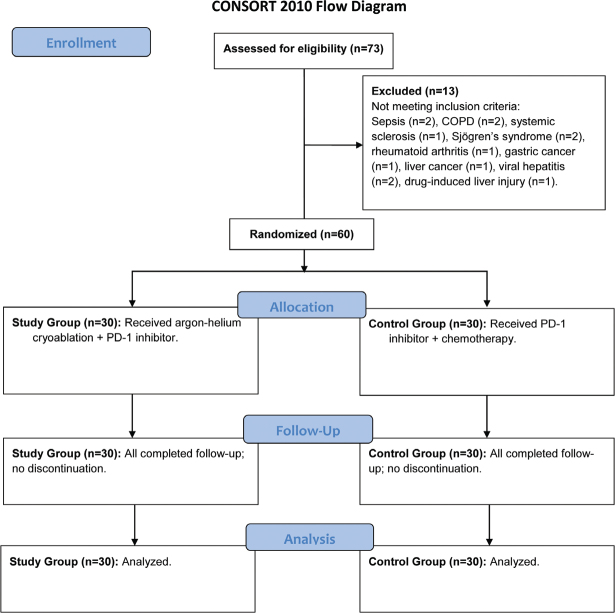
Kaplan–Meier Curves for Overall Survival (OS). The curve for the study group (Argon–Helium Cryoablation + PD-1 inhibitor) shows a higher survival probability over time compared to the control group (Chemotherapy + PD-1 inhibitor). The median OS was not reached in the study group versus 10.3 months in the control group (*P* = 0.003 by log-rank test).

**Figure 3:**
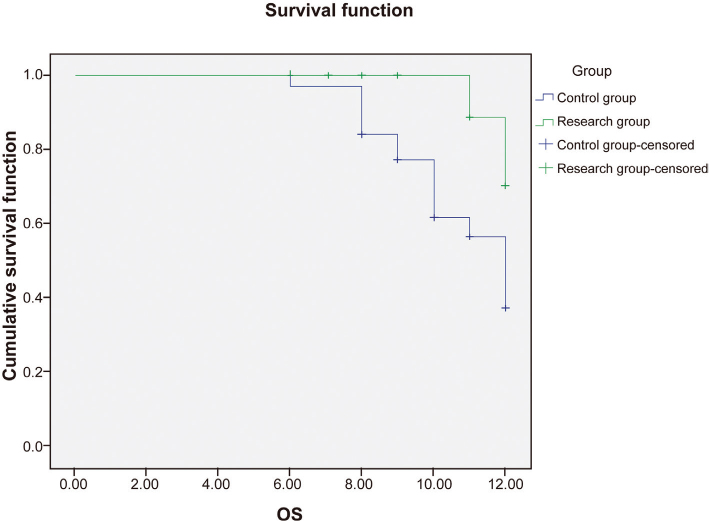
Kaplan–Meier Curves for Progression-Free Survival (PFS). The curve for the study group shows a longer time to progression or death compared to the control group. The median PFS was 9.6 months in the study group versus 8.3 months in the control group (*P* = 0.005 by log-rank test).

The median follow-up duration for all surviving patients was 12.8 months. At the 1-year follow-up, the PFS rate, as estimated by the Kaplan–Meier method, in the study group was 63.3% (95% CI: 46.1–80.5%), compared to 43.3% (95% CI: 25.5%–61.1%) in the control group. Kaplan–Meier analysis revealed that the study group had a statistically significantly better OS (median not reached vs. 10.3 months; HR 0.45, 95% CI: 0.24–0.83; *P* = 0.003). Similarly, the study group had statistically significantly longer median PFS (9.6 months vs. 8.3 months; HR 0.48, 95% CI: 0.27–0.85; *P* = 0.005) (Figures 2 and 3).

## Discussion and conclusion

The advent of ICIs, particularly PD-1/PD-L1 inhibitors, has significantly advanced the treatment paradigm for NSCLC, with monotherapy or combination regimens becoming the standard of care [11, 23]. Camrelizumab, the PD-1 inhibitor used in this study, targets the PD-1 receptor, disrupting its interaction with PD-L1/PD-L2 and restoring anti-tumor T-cell activity [13]. For instance, the pivotal CameL study demonstrated long-term OS benefits with camrelizumab plus chemotherapy, but a substantial proportion of patients do not achieve durable responses, highlighting the need for novel combination strategies [19, 24]. Our study explores whether augmenting systemic immunotherapy with local cryoablation can overcome some of these limitations.

Regarding long-term outcomes, our study demonstrated statistically significantly prolonged OS and PFS in the argon-helium cryoablation plus PD-1 inhibitor group. The magnitude of improvement in median PFS (1.3 months) and the substantial benefit in OS, where the median was not reached in the study arm, are considered clinically meaningful in this advanced disease setting. This survival benefit, particularly the marked improvement in OS, suggests that the combination of local tumor destruction via cryoablation and systemic immune potentiation offers a durable clinical advantage over standard chemo-immunotherapy. Our results build upon findings from earlier, non-randomized studies and provide robust, controlled evidence supporting this combination as a highly promising therapeutic strategy for advanced NSCLC [25, 26]. The median OS benefit observed in our study compares favorably with historical data from chemo-immunotherapy trials, suggesting that this combination not only extends survival but may also offer a new standard for patients suitable for local ablative therapy. While quality of life was not a formal endpoint, the minimally invasive nature of cryoablation may contribute to better patient tolerance compared to repetitive, systemic chemotherapy cycles.

A key finding of our research is the statistically significant improvement in immune function parameters in the study group. Tumor immune escape is a critical aspect of cancer progression, with lymphocytes playing a pivotal role [27]. CD4+ T helper cells orchestrate immune responses, while CD8+ CTLs directly kill tumor cells. The CD4+/CD8+ ratio reflects the balance of cellular immunity and is an indicator of immune status; an increased ratio generally suggests enhanced immune function [28, 29]. Our results showed that after treatment, patients receiving argon–helium cryoablation plus a PD-1 inhibitor had statistically significantly higher CD4+ counts and CD4+/CD8+ ratios, and lower CD8+ counts compared to the chemo-immunotherapy group. This suggests a more favorable immunomodulatory effect. The reduction in peripheral CD8+ T cells might reflect increased trafficking and infiltration of activated CTLs into the tumor microenvironment, or a reduction in exhausted/dysfunctional CD8+ T cells, rather than overall depletion [30]. Cryoablation-induced tumor antigen release may enhance priming and activation of tumor-specific T cells, which are then further potentiated by PD-1 blockade [14, 31]. This is consistent with studies showing that effective anti-tumor therapy can reverse immune escape and normalize lymphocyte subset distributions [32]. Li et al. [33] also reported enhanced immune function after cryoablation in lung cancer patients.

In our study, while the ORR and DCR were numerically higher in the argon–helium cryoablation plus PD-1 inhibitor group, the differences did not reach statistical significance. This finding might be attributed to the relatively small sample size, which was powered for survival endpoints rather than response rates, potentially lacking sufficient power to detect modest differences in ORR/DCR.

The safety profile of argon–helium cryoablation combined with PD-1 inhibitors was comparable to that of PD-1 inhibitors plus chemotherapy, with no new or unexpected severe AEs. This indicates that adding cryoablation to PD-1 inhibitor therapy does not significantly increase treatment-related toxicity, which is crucial for its clinical applicability, especially in patients who may be frail or have comorbidities [34, 35]. This profile suggests that the AEs associated with the combination are largely predictable and manageable with standard supportive care and established irAE management algorithms. Prophylactic monitoring, including regular pulmonary function tests and liver function monitoring, can facilitate early detection and intervention, further enhancing the clinical applicability of this regimen.

This study has several limitations. Firstly, it was a single-center study with a relatively small sample size, which may limit the generalizability of the findings and the statistical power to detect smaller differences in some endpoints, despite being powered for primary survival outcomes. Secondly, the open-label design could introduce performance bias, although outcome assessment for major efficacy endpoints was blinded. Thirdly, the follow-up period was limited to 1 year, and longer-term follow-up is necessary to fully assess the durability of the observed benefits and detect late-onset AEs. Fourthly, while we observed changes in peripheral blood immune cell subsets, a more detailed analysis of the tumor microenvironment, including tumor-infiltrating lymphocytes and PD-L1 expression dynamics, would provide deeper insights into the mechanisms of action. Biomarker analyses to predict response would also be valuable. Finally, the choice of chemotherapy backbone in the control arm, while reflecting real-world practice, adds minor heterogeneity.

In conclusion, argon–helium cryoablation combined with PD-1 inhibitors demonstrated promising efficacy in patients with NSCLC, characterized by statistically significant improvements in OS, PFS and favorable modulation of systemic immune function, when compared to PD-1 inhibitors plus standard chemotherapy. The short-term response rates were numerically higher, though not statistically significant. Importantly, this combination therapy did not result in increased AEs and showed a manageable safety profile. These findings suggest that argon–helium cryoablation plus PD-1 inhibitors represents a viable and potentially superior treatment option for selected NSCLC patients, meriting further investigation in larger, multi-center randomized controlled trials.

## Supplementary Material



## Data Availability

The datasets generated and analyzed during the current study are available from the corresponding author on reasonable request.
