# Supplementary material for: Efficacy of argon–helium cryoablation combined with PD-1 inhibitors in non-small cell lung cancer
Source: Acta Oncol. 2025 Nov 12;64:44215. doi: 10.2340/1651-226X.2025.44215 (PMC12625152; doi:10.2340/1651-226X.2025.44215)

Supplementary material has been published as submitted. It has not been copyedited, or typeset by Acta Oncologica

Supplementary figure 1: **CONSORT Flow Diagram of Patient Enrollment.**

A total of 73 patients were assessed for eligibility. Thirteen were excluded due to predefined criteria (sepsis, comorbidities, or other malignancies). Sixty patients were randomized into the study group (argon-helium cryoablation + PD-1 inhibitor) or control group (chemotherapy + PD-1 inhibitor). All participants received allocated interventions and completed follow-up, with no attrition.

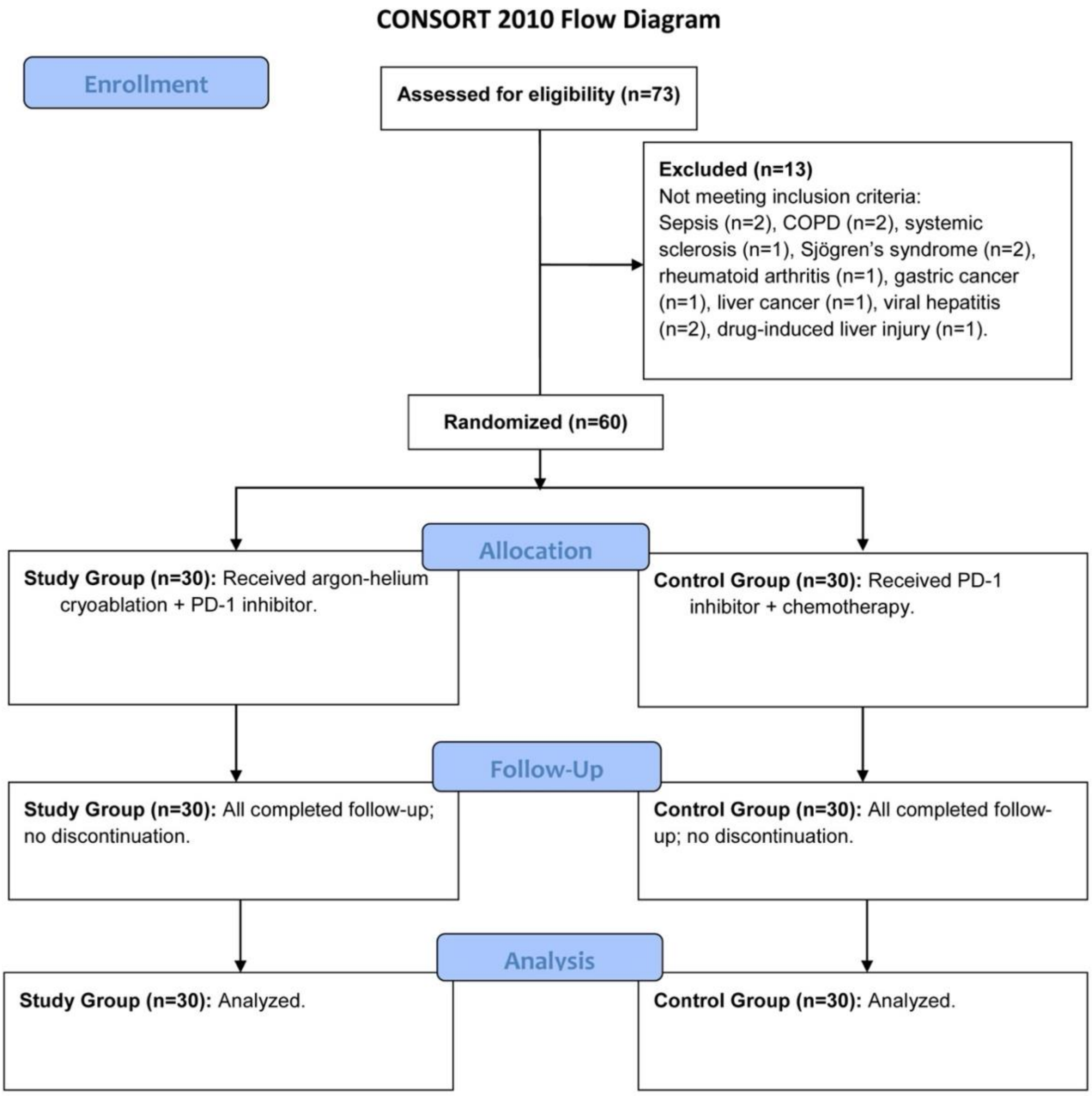

Supplement: Supplementary file 1 [file AO-64-44215-s1.pdf]
